# Evaluation of Differential Gene Expression during Transdifferentiation of Bone Marrow Stromal Cells to Glial Phenotype in the Presence of Cerebrospinal Fluid

**Published:** 2019

**Authors:** Hatef Ghasemi Hamidabadi, Maryam Nazm Bojnordi, Nourollah Rezaei, Sara Soleimani

**Affiliations:** 1. Immunogenetic Research Center, Department of Anatomy and Cell Biology, Faculty of Medicine, Mazandaran University of Medical Sciences, Sari, Iran; 2. Department of Anatomy & Cell Biology, Faculty of Medicine, Hamadan University of Medical Sciences, Hamadan, Iran

**Keywords:** Bone marrow stromal cells, Cells, Cerebrospinal fluid, Oligodendrocyte, Oligoprogenitor

## Abstract

**Background:**

The present study assessed the alteration of gene expression during transdifferentiation of Bone Marrow Stromal Cells (BMSCs) into oligodendrocyte in the presence of Cerebrospinal Fluid (CSF).

**Methods:**

BMSCs were collected from female Sprague-Dawley rats and were cultured in DMEM/F12 medium supplemented with Retinoic Acid (RA), basic Fibroblast Growth Factor (bFGF), and Epidermal Growth Factor (EGF). CSF was added daily to the culture media. The oligoprogenitor and oligodendrocyte generation was assessed by immunocytochemistry for Oligo 2, A2B5, CNP and MBP markers.

**Results:**

The mean percentages of immunopositive cells for Olig2 and A2B5 were 52.1±1.74 and 56.34±2.55%, respectively. The number of immunopositive cells for glial markers CNP and MBP were 48.8±3.12 and 40.5±8.92%, respectively. Alteration of gene expression of Oct4, Olig 2, PDGFR-*α* and PLP were examined by real time PCR during transdifferentiation of BMSC to oligodendrocyte. Immunocytochemical results indicate that oligoprogenitor cells were immunopositive for Oligo2 and A2B5 markers. Also, oligodendrocytes expressed the mature glial markers of CNP and MBP indicating successful differentiation.

**Conclusion:**

In conclusion, CSF promotes the transdifferentiation of BMSC into mature oligodendrocyte via providing an appropriate niche for glial maturation.

## Introduction

Myelin sheath of neural fibers is synthesized by oligodendrocytes that conduct the nerve impulses in central nervous system 1. Disruption in myelin sheat structure may cause neural dysfunction, a prevalent symptom in demyelinating disease such as multiple sclerosis 2,3. Cell therapy focused on generation of oligodendrocytes has the positive effect on myelin repair of demyelinated nerves 4–6. In total, the remyelination facilitated transplantation of cells with some criteria such as property of migration and myelinogenic capacity after injection into damaged areas 7,8. Therefore, providing methods for *in vitro* enrichment of oligodendrocytes is a helpful step in cell therapy of demyelinating disease 9,10. Some reports show generation of oligodendrocyte from various stem cell types. It seems Bone Marrow Stromal Cells (BMSCs) are an appropriate choice because of their property of *in vitro* derivation into different cell types including neuron and glia cells 11,12. Improvement of differentiation capacity of BMSC into oligodendrocytes eliminates the limitation of sufficient oligodendrocyte generation. To this aim, several groups used different induction protocols and various inducers in order to get differentiation of BMSC to oligodendrocytes 13–15.

Recently, Cerebrospinal Fluid (CSF) has been used as an inducer for *in vitro* neural induction. It seems CSF may provide an appropriate environment that stimulates differentiation *via* secretion of growth and neurotrophic factors 16,17. Since CSF is a complex fluid containing many components, *i.e*. essential growth and survival factors, CSF was tested and an efficient culture system was designed by adding CSF to a cocktail of inducers. Our culture system is usable as an alternative procedure which leads to significant enhancement in generation of oligodendrocyte from BMSC. The aim of this research was to evaluate differential gene expression during transdifferentiation of BMSC into oligodendrocytes in the presence of CSF.

## Materials and Methods

### Collection and culture of BMSC

Animal research was performed after approval by the animal ethics committee of Mazandaran University of Medical Sciences. After euthanasia of Sprague-Dawley female rats (6 to 8 weeks old weighing 250 to 300 *g*), bone marrow cells were collected from femurs by flushing the shaft with a needle and syringe using saline. Five rats in each experiment with eight replicates were used. These cells were disassociated, then washed and centrifuged for 10 *min* at 1500 *rpm*. The cells were cultured in Dulbecco's Modified Eagle Medium (DMEM) (Invitrogen Corp., Carlsbad, CA, USA) supplemented with 10% Fetal Bovine Serum (FBS) (Invitrogen) in a 37°*C* incubator with 5% CO_2_. Culture medium was replaced every 2 days. When fibroblast like cells attached onto the base of culture plates reached confluence, they were trypsinized using 0.25% trypsin/EDTA (Gibco, Thermo Fisher Scientific, North Nelson Industrial Estate, UK) and then passaged. This was considered the first passage (P1) followed by three additional passages 18,19.

### Collection and preparation of CSF

Fifty rat embryos were used for each experiment. Cerebrospinal fluid was collected from cisterna magna of 19 day embryos and was transferred into sterile micro tubes then centrifuged at 1200 *rpm* and finally stored in −20*°C* freezer. This final preparation included DMEM medium with 5% CSF 20.

### Morphological characterization of undifferentiated BMSC

Cell growth and morphological features of the undifferentiated cells were examined and photographed daily using phase contrast microscopy (Olympus inverted phase contrast microscope (BX51), Olympus, Tokyo, Japan). Morphological changes of cells during induction stage were monitored daily and glial differentiation was confirmed during the induction phase.

### Multilineage differentiation

The multipotency of BMSC was evaluated *via* differentiation into osteoblast and adipocyte according to standard staining protocols *e.g*. alizarin red for calcium and oil red for lipid. The treated cells were stained with Alizarin Red/Oil Red Staining Solution (#TMS-008-C, Sigma, St. Louis USA). Osteogenic differentiation medium included DMEM containing 10 *nM* β-glycero-phosphate, 80 *μg/ml* ascorbic acid and 10 *nM* dexamethasone. Adipogenic differentiation medium consisted of DMEM with 10% FBS and 100 *nM* dexamethasone.

### Flow cytometry

To perform flow cytometry, BMSCs from the fourth passage (P4) were trypsinized, washed and resuspended in PBS. All antibodies were obtained from Abcam. Cells were stained for mesenchymal cell surface marker antibodies, CD90, CD44 and CD73. Cells were also stained for hematopoietic stem cell markers; CD33 and CD34. All antibodies were incubated for 30 *min* at room temperature in a dark place. The stained cells were then washed with PBS and then incubated with appropriate secondary antibodies for 60 *min* at room temperature in a dark place.

The flow cytometry results were analyzed using a FACS Calibur™ (BD Biosciences, Singapore). The flow cytometry results were analyzed with Cellquest™ WinMDI software (BD Biosciences).

### Differentiation of BMSC into oligodendrocyte

After the fourth passage (P4), BMSCs were cultured in DMEM/F12 media (Sigma-Aldrich) supplemented with 5% FBS, 1 *μM* Retinoic Acid (RA) (Sigma-Aldrich), 20 *ng/ml* basic Fibroblast Growth Factor (bFGF) supplement (Sigma-Aldrich) and 10 *ng/ml* Epidermal Growth Factor (EGF) (Sigma-Aldrich) for 2 days in order to have induction of oligoprogenitor cells. This treatment was followed by induction with CSF for 8 days to obtain oligodendrocytes. CSF was added to the culture medium every day and the differentiated cells were examined daily during the induction protocol. Three control groups were included as follows: Control 1: BMSC from P4 cultured in DMEM/F12/5% FBS for 10 days, Control 2: BMSC from P4 cultured in DMEM /F12/5% FBS/RA/EGF/bFGF for 10 days and, Control 3: BMSC from P4 cultured in CSF for 10 days.

### Immunocytochemical preparation for fluorescence microscopy

To identify the differentiated oligoprogenitor cells and oligodendrocytes, immunocytochemistry technique was done. The cells were fixed in 4% paraformaldehyde (pH=7.4) followed by incubation in 5% goat serum blocking buffer for 30 *min*. All antibodies were from Abcam, Milton, Cambridge, UK. Immunostaining was performed for oligoprogenitor cells and oligodendrocyte using Olig2 and A2B5 antibodies for oligoprogenitor cells, CNP and MBP antibodies for mature oligodendrocytes. Antibodies were diluted in PBS and incubated at 4°*C* overnight then rinsed twice with PBS buffer. Cells were then incubated with secondary antibodies for 60 *min* at room temperature in a dark place, rinsed with PBS, and counterstained with DAPI 1:1000 (Sigma-Aldrich) for 1 *min*. Sections were cover slipped with 90% glycerol. The stained cells were observed under fluorescent microscope (Leica, Wetzlar, Germany).

### Real time polymerase chain reaction (Real timePCR)

To investigate differentiated oligoprogenitor cells and oligodendrocytes, real time PCR was performed to evaluate the expression of oligoprogenitor and mature oligodendrocyte markers. *Olig2*, *PDGF-α* and *PLP* genes were evaluated. Gene expression alteration was monitored during *in vitro* differentiation of BMSC into oligoprogenitor and oligodendrocyte cells.

Total RNA was extracted from BMSC, oligoprogenitor and oligodendrocyte cells using RNX Plus TM (Cinnagen, Iran). The cDNAs were synthesized using cDNA Synthesis Kit (Fermentas, Lithuania). PCR was carried out using Master Mix and SYBR Green (Applied Biosystems, Foster City, CA, USA) in a thermocycler (Applied Bio-systems). The PCR program started with an initial melting cycle, 4 *min* at 94°*C*, to activate the polymerase followed by 40 cycles of a melting step (20 *s* at 94°*C*), an annealing step (30 *s* at 57°*C*), and an extension step (30 *s* at 72°*C*). Primer sequences (forward and reverse) were as follows: *Oct4* gene (mulipotency marker of BMSC) was assessed using the 5′ AAGCTGCTGAAACAGAAGAGG 3′ forward primer and the 5′ ACACGGTTCTCAATGCTAGTC 3′ reverse primer. The expression of rat *PDGFR-α* gene (oligoprogenitor marker) was assessed using forward 5′CTAATTCACATTCGGAAGGTTG 3′ and reverse 5′GGACGATGGGCGACTAGAC 3′. The expression of *Olig2* gene (oligoprogenitor marker) was evaluated with forward 5′ CCGAAAGGTGTGGATGCTTAT 3′ and reverse 5′ TCGCTCACCAGTCGCTTCAT 3′. Expression of *PLP* gene, the marker for mature oligodendrocyte, was done using forward primer 5′ AGTC TGTGTCTGGAGAGCAG 3′ and 5′ TACGGTATCG CCGCTCCCGATTCGCA-3 the reverse primer. The expression of *β actin* gene (positive control) was evaluated using 5′ CTTCTTGGGTATGGAATCCTG 3′ forward primer and 5′ GTGTTGGCATAGAGGTCTT TAC 3′ reverse primers 11–13. The data were evaluated using StepOne software V2.1 (Applied Biosystems). Gene expression levels were calculated according to 2^−ΔΔ*Ct*^ formula.

### Statistical analysis

One-way analysis of variance (ANOVA) and Tukey post hoc test (SPSS 13.0 software) were used for analyzing the data (p<0.05 was considered significant).

## Results

### Characterization of BMSC

**Figure 1.**
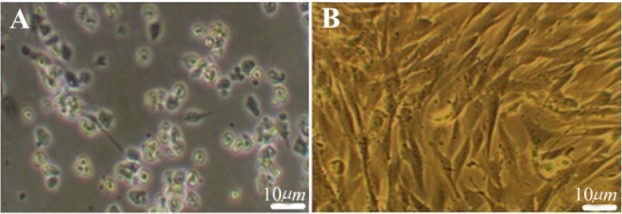
The morphological appearance of cultured BMSC. A) Primary culture of BMSC after 4 *hr* culture, B) The cell population after the 4th passage culture. Scale bars 10 *μm.*

**Figure 2.**
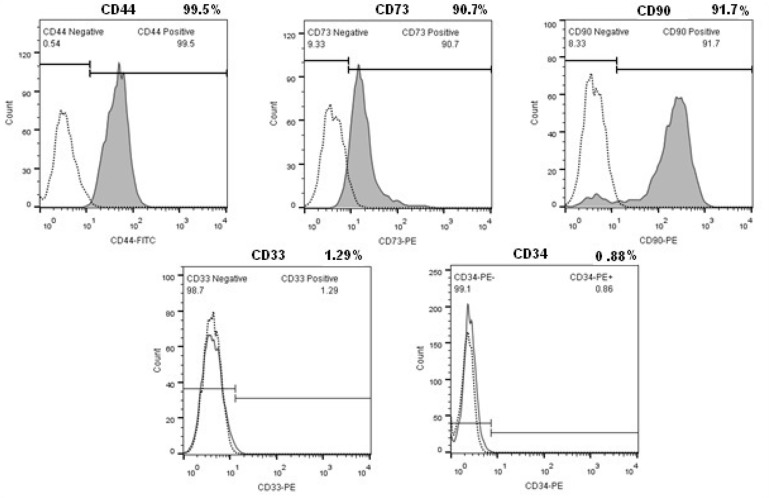
Flow cytometric analysis of BMSC (Passage 4). Isolated bone marrow cells are strongly positive for MSC surface markers but didn’t express CD33 and CD34.

**Figure 3.**
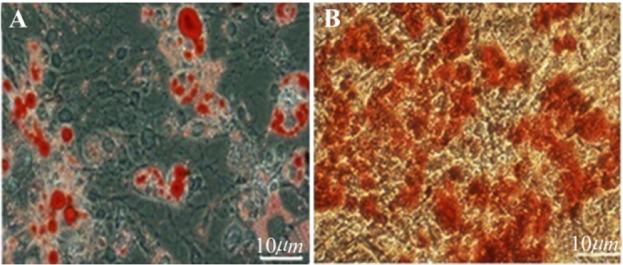
Multilineage differentiation of BMSC, A) Adipogenic differentiation of BMSC (Oil Red O stain of lipid droplets). B) Osteogenic differentiation of BMSC; (Alizarin Red Stain for calcium). Scale bars 10 *μm.*

The morphological evaluation showed the proliferative and adherent activity of the isolated BMSC. Bone marrow stromal stem cells had spindle shaped morphology similar to fibroblast morphology and were attached tightly to the flask (Figure 1). This morphological appearance confirmed the mesenchymal characteristics of BMSC. The flow cytometry results showed BMSCs were positive for the mesenchymal markers; CD90, CD44 and CD73 but were negative for CD33 and CD34 hematopoietic markers (Figure 2). Multilineage differentiation was evaluated to confirm the MSC characteristics of isolated cells. Adipogenic differentiation was confirmed by oil red O staining of lipid droplets (Figure 3A) and osteogenic differentiation by production of calcium deposits stained with alizarin red (Figure 3B).

### Differentiation of oligoprogenitor cells from BMSC

**Figure 4.**
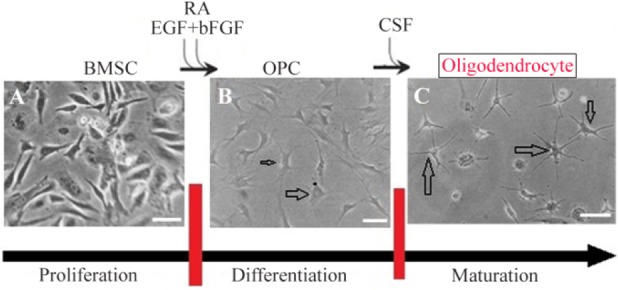
Scheme showing the protocol for differentiation of BMSC to oligodendrocyte. For glial cells, undifferentiated BMSCs A) were plated in media that induce OPC generation B). Further cultivation in the presence of CSF was used to complete the maturation of OPC to Oligodendrocyte C). Scale bars 10 *μm.*

**Figure 5.**
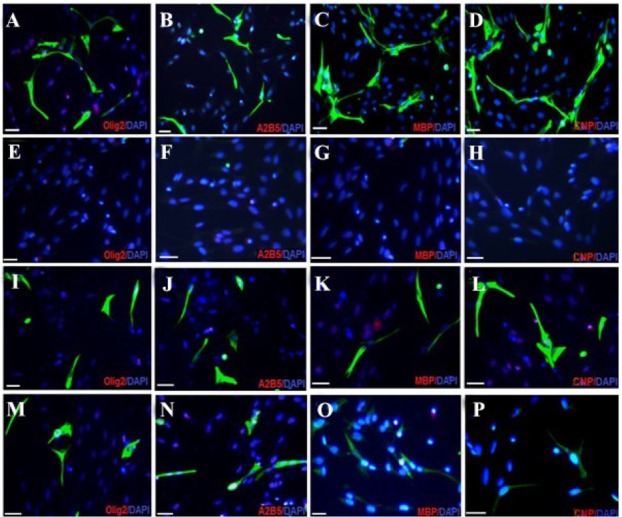
Immunocytochemistry analysis of differentiated bone marrow stromal cells into oligodendrocyte like cells. Fluorescence images of oligoprogenitor markers (Olig2 and A2B5) and mature oligodendrocyte markers; (CNP and MBP) in experimental group (A-D); Negative Control 1 (E-H) Control 2 (I-L), and Control 3. (M-P). Scale bars 10 *μm.*

**Figure 6.**
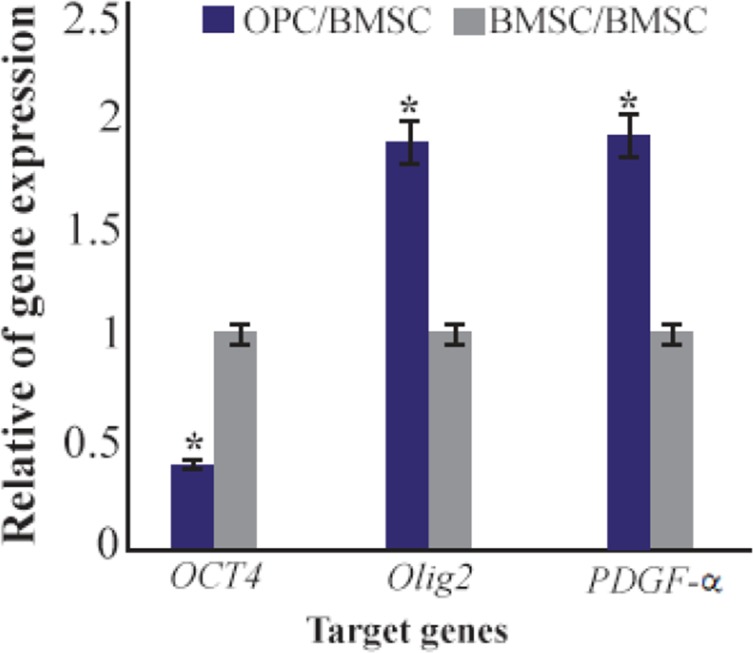
Ratio of gene expression of *Oct4*, *Olig2*, *PDGF-α* in oligoprogenitor cells compared to BMSC. *: Significant increase or decrease. OPC: Oligoprogenitor cells. BMSC: Bone marrow stromal cells. Data are shown as mean±SEM from three independent experiments.

Oligoprogenitor cells were induced in the presence of CSF followed by differentiation to an oligodendrocyte structure. Morphological changes that appeared in cells exposed to CSF were characterized by phase contrast microscopy (Figures 4B and 4C). Differentiation into oligoprogenitor cells was done by immunostaining with Olig2 and A2B5 which confirmed these glial progenitor markers (Figures 5A and 5B). The mean percentages of immunopositive cells for Olig2 and A2B5 were 52.1±1.74 and 56.34±2.55%, respectively. Figure 6 compares *Oct4*, *Olig2* and *PDGF-α* genes expressions obtained by real time PCR. The expression of *Olig2* and *PDGF-α* genes significantly increased in oligoprogenitor cells as compared to untreated BMSC. The expression of Oct4 was down-regulated during transition of BMSC to oligoprogenitor cells (Figure 6).

### CSF increases differentiation of mature oligodendrocyte

At the end of the induction phase, glial progenitor-like cells were developed into a branched morphology. The glial appeared as multi-processes (Figure 4C). After exposure to CSF, immunostained cells were dissociated for expression of the specific markers, CNP and MBP, to identify mature oligodendrocytes. The proportion of immunostaining results showed that at the end of the induction protocol, the differentiated cells were immunopositive for the mature oligodendrocyte markers; CNP and MBP (Figures 5C and 5D).

Also no positive reaction for glial markers was detected in Control 1 which received no treatment (Figures 5E–H). The number of immunopositive cells for glial markers (Olig2, A2B5, CNP and MBP) in Control 2 were 49.8±4.64, 47.33±7.84, 48.8±3.12, 40.5±8.92, respectively and in Control 3 were 31.40±2.12, 30.13± 5.39, 25.5±2.98, 29.17±9.30%, respectively (Figure 5; I–L; M–P).

**Figure 7.**
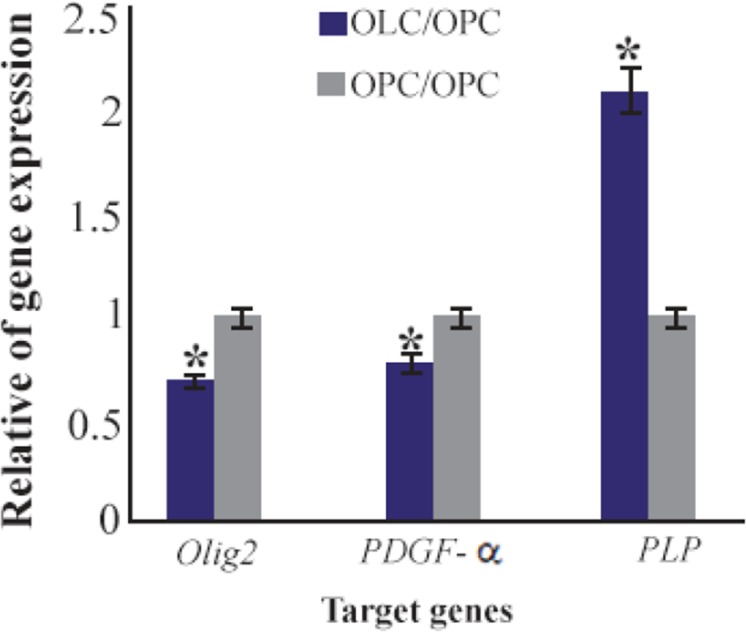
Ratio of gene expression of *Olig2, PDGF-α* and *PLP* in oligodendrocyte compared to oligoprogenitor cells. *: Significant increase or decrease. OPC: Oligoprogenitor cells. OLC: Oligodendrocyte. Data are shown as mean ±SEM from three independent experiments.

**Figure 8.**
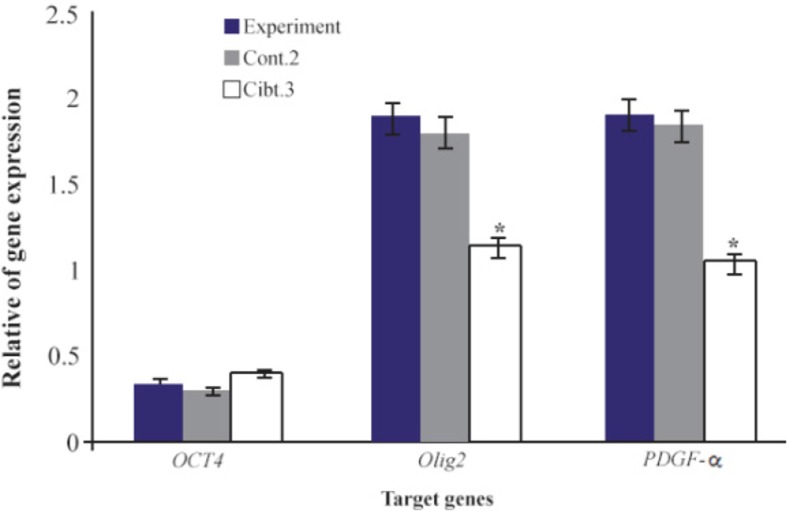
Comparison of gene expression of *Oct 4*, *Olig2*, *PDGF-α* in experimental group compared to Control 2 and Control 3. *: Significant decrease with experimental group. Data are shown as mean± SEM from three independent experiments.

Data of real time PCR showed a significant increase in expression of PLP in mature differentiated oligodendrocytes at the end of our induction protocol as compared to oligoprogenitor cells (Figure 6). Olig2 and PDFG-*α* were down regulated in differentiated oligodendrocytes as compared to oligoprogenitor cells (Figure 7). The glial markers, Olig2, PDGF-*α* and PLP, were expressed more in the experimental group as compared to Control 2 and Control 3 (Figure 8). Data are shown as mean±SEM from three independent experiments.

## Discussion

BMSC has a therapeutic application in tissue engineering and for treatment of neurodegenerative disease because of BMSC ability to differentiate into various neural cell types 18,19. This indicates cell therapy techniques based on *in vitro* BMSC differentiation which is an effective treatment of a central nervous system lesions 19,20. *In vitro* generation of oligodendrocytes could be usable as a source for cell therapy.

Derivation of high pure population of oligodendrocytes is a controversial issue that needs some specific conditions. This process is possible *via* various differentiation protocols using different inducers and growth factors to mediate cell differentiation. In this study, differentiated BMSC was directed to become oligodendrocytes and among various protocols mentioned, CSF was selected for the induction of differentiation.

Cerebrospinal fluid is a mixture of various nutrients, growth factors and an extracellular matrix component that have directly interacted with stem cell niches of neural system. Growth factor [CSF-Insulin-Like Growth Factor-2 (IGF2)] stimulates brain development. CSF-IGF2 controls cell division *via* specific surface receptors of neural stem cells. Specific key morphogens elements such as FGF-2, EGF, and retinoic acid recognized as a survival and mitogenic factor for oligodendrocyte progenitor cells promote glial differentiation protocol that influences neurogenesis 21,22. The CSF complex of factors, nutrients and components mediate the maturation process of undifferentiated cells 21,22. Differentiated BMSCs were promoted to oligodendrocytes using CSF as an inducer that prepares a microenvironment similar to the natural conditions of the central nervous system.

At the first step, the morphological features of BMSC were investigated. This is a spindle shaped cell population with a high proliferation and adherent activity similar to the results from previous reports 23–26. The morphological feature changes of differentiated oligoprogenitor were monitored and oligodendrocyte was evaluated *via* phase contrast microscopy. In this research, adding CSF to the medium increased differentiation of oligoprogenitor cells as well as their maturation. The results of this research are similar to a finding in which the promotional role of CSF in differentiation of neural progenitor was proved 27.

Oligoprogenitor cells with protruding processes were differentiated into mature oligodendrocyte cells after 8 days of incubation with CSF. Generation of mature oligodendrocyte with the ability of remyelination could be used as a therapeutic approach to repair demyelination diseases.

Immunocytochemistry dates showed that BMSC exposed to supplements of RA, bFGF and EGF expressed glial markers such as Olig2 and A2B5, which have no expression in the untreated BMSC (Control 1). These results are in agreement with previous results reporting glial markers in undifferentiated BMSC 14,15.

At the end of the differentiation protocol and in the presence of CSF, the differentiated BMSCs had positive reaction for CNP and MBP (markers of mature glial cells) while the undifferentiated BMSCs didn’t express these markers. The differentiated oligodendrocytes expressed more functionality markers, CNP and MBP, upon exposure to CSF fluid in comparison to Control 2 which didn’t receive any CSF.

Our protocol was based on using natural, organic material *e.g*. cerebrospinal fluid, and led to derivation of oligodendrocytes compared to previous research studies using only chemical inducers 23,24. Those research studies differentiated BMSCs to oligodendrocytes using some inducers including bFGF, PDGF and T3. The low presence of mature oligodendrocytes in this type of research is related to using chemical inducers that are potentially pernicious and can damage the cells over long periods of time during culture.

It seems that CSF as used in our protocol can mediate glial differentiation and maturation *via* activation of a subset of genes involving myelinogenesis. Transition of BMSC to oligodendrocytes is accompanied with a wide range of gene expression changes. Our real time PCR date showed over expression of *Olig2* and *PDGF-α* genes in oligoprogenitor cells as compared with untreated BMSC. Also, the expression of the pluripotent marker Oct4 was down-regulated during transition of BMSC to oligoprogenitor cells. The maturation of oligoprogenitor cells was completed in the presence of CSF using detection of the *PLP* gene.

PLP marker was expressed in mature oligodendrocytes while this marker has no expression in untreated BMSC and oligoprogenitor cells. In addition, *Olig2* and *PDGF-α* genes were down regulated in mature oligodendrocytes as compared to oligoprogenitor cells. The results support the previous reports about down regulation of these genes in mature oligodendrocytes. Also, the up regulation of PLP was the evidence which confirmed the differentiation of mature oligodendrocytes involved in myelin synthesis using our induction system supplemented with CSF.

In two previous studies, the successful rate of derivation of oligodendrocytes from BMSC was at a low level 14,15. It seems the inducers used in these research studies affected only a limited number of stem cells. However, the division neurotrophic effect of CSF components, such as CSF-insulin-like growth factor-2 (IGF2), EGF, FGF-2 and RA acts as a survival and mitogenic factor for oligodendrocyte progenitor cells and promotes glial differentiation protocol. Since CSF has the enriched microenvironment including various growth factors, extracellular matrix elements and nutrient components, it can affect the neural stem cell niches 23–25. CSF has the components that mediate proliferation of neural stem cells and complete the maturation of the progenitor cells 28,29. It seems the addition of CSF into the differentiation medium facilitates differentiation of BMSC to oligodendrocyte phenotype *via* providing a suitable microenvironment similar to a natural neural condition 30,31. CSF increases development of oligoprogenitor cells and mediates signaling pathways *via* cellular receptors and gene expression including EGF and BDNF receptors 32,33.

## Conclusion

In our research, the results indicated CSF can promote differentiation of BMSC into oligodendrocytes. The differentiated oligodendrocytes using our differentiation protocol could be used for cell therapy in neurodegenerative disorders. Differentiation of oligodendrocytes from BMSC is also a possible therapeutic approach in regenerative medicine for remyelination of damaged nerve fibers.
